# Neurophysiological and Behavioral Responses to Gambling Cues in Individuals with Internet Gambling Disorder: A VR-EEG-GSR Study

**DOI:** 10.3390/brainsci16080832

**Published:** 2026-08-05

**Authors:** Selami Varol Ülker

**Affiliations:** Neuro PsychoPhysiology Research Laboratory, Psychology Department, Faculty of Humanities and Social Sciences, Üsküdar University, Istanbul 34662, Türkiye; selamivarol.ulker@uskudar.edu.tr

**Keywords:** internet gambling disorder, cue reactivity, virtual reality, EEG, GSR, P300, LPP, skin conductance, craving, behavioral addiction

## Abstract

**Highlights:**

**What are the main findings?**

- Individuals with Internet Gambling Disorder exhibited enhanced P300 and late positive potential (LPP) responses, increased skin conductance, and greater subjective craving during exposure to gambling-related cues in an immersive virtual reality environment.
- Near-miss gambling outcomes elicited the strongest emotional and autonomic responses, supporting their role as particularly salient and reinforcing cues in problematic online gambling.

**What are the implications of the main findings?**

- Combining virtual reality with EEG and galvanic skin response provides an ecologically valid, multimodal approach for investigating cue reactivity in behavioral addictions.
- The present findings suggest that neurophysiological and autonomic measures may provide complementary information regarding cue reactivity in Internet Gambling Disorder. Additional studies with larger samples and longitudinal designs are needed before these measures can be considered for clinical application.

**Abstract:**

Background: Internet Gambling Disorder (IGD) has become an important public health concern, yet relatively little is known about the combined neural, autonomic, and behavioral mechanisms underlying cue reactivity in immersive gambling environments. This study investigated whether individuals with IGD demonstrate altered neurophysiological and psychophysiological responses during a virtual reality (VR) gambling task. Methods: Fifty-seven adults, including 28 participants meeting diagnostic criteria for IGD and 29 healthy controls, completed a standardized VR casino paradigm incorporating predetermined win, loss, and near-miss events. Electroencephalography (EEG), galvanic skin response (GSR), behavioral performance, and post-task craving were assessed simultaneously. Results: Compared with healthy controls, the IGD group demonstrated significantly greater P300 amplitudes during win trials and enhanced late positive potential (LPP) responses following near-miss outcomes, indicating increased attentional allocation and sustained emotional processing of gambling-related cues. Skin conductance responses were also significantly higher in response to near-miss events, whereas overall skin conductance level and reaction time did not differ significantly between groups. Participants with IGD additionally exhibited greater engagement during the VR task and reported substantially higher craving following exposure to the gambling environment. Conclusion: These findings indicate that immersive VR combined with EEG and autonomic recordings provides a sensitive framework for detecting cue-reactivity associated with IGD. Although the findings should be interpreted in light of the relatively modest sample size and the use of a predominantly male sample, they suggest that immersive VR combined with EEG and autonomic recordings provides a sensitive framework for investigating cue reactivity associated with Internet Gambling Disorder. Integrating behavioral, physiological, and neural measures may improve understanding of the mechanisms underlying problematic gambling and inform future research on objective assessment strategies and intervention development.

## 1. Introduction

### 1.1. Background

Gambling Disorder is recognized in both the DSM-5 and DSM-5-TR as a Substance-Related and Addictive Disorder and remains the only behavioral addiction formally included alongside substance use disorders [1]. This designation reflects the disorder’s clinical significance and highlights important similarities with substance addictions, including persistent compulsive behavior, diminished control over gambling, progressive increases in gambling involvement resembling tolerance, and emotional distress that resembles withdrawal symptoms when gambling is interrupted [2]. Despite considerable adverse effects on financial stability [3], occupational functioning [4], interpersonal relationships [5], and psychological well-being [6], many individuals continue to gamble. In most cases, gambling-related problems emerge gradually, evolving from recreational participation into a persistent and compulsive pattern that becomes increasingly difficult to control voluntarily [7].

The development and maintenance of Gambling Disorder involve multiple interacting psychological processes, including maladaptive gambling-related beliefs such as the illusion of control and the gambler’s fallacy [8], disturbances in emotional regulation [9], and heightened impulsivity during decision-making [10]. Together, these factors promote repeated risk-taking and persistent loss-chasing behaviors [11], reinforcing the addictive cycle. Although conventional forms of gambling remain common, internet-based gambling has become increasingly prevalent [12]. Online gambling environments differ substantially from traditional venues by providing continuous accessibility, rapid betting opportunities, and highly interactive experiences that may intensify craving, cue reactivity, and deficits in executive control. Consequently, understanding the psychopathological mechanisms underlying modern digital gambling has become increasingly important as gambling technologies continue to evolve.

### 1.2. Rise of Internet-Based Gambling

In recent years, internet-based gambling has emerged as a dominant modality [13], especially among younger populations [14]. The increasing ubiquity of smartphones [15], online casinos [16], and sports betting platforms [17] has dramatically changed the landscape of gambling behavior. Unlike traditional in-person gambling venues, online platforms offer 24/7 access, anonymity, fast-paced betting cycles, and the ability to gamble across multiple games simultaneously. These features are particularly potent in reinforcing problematic gambling patterns, as they provide continuous exposure to gambling cues and minimize natural barriers such as time, location, and social scrutiny.

Online gambling is also associated with greater difficulty in regulating behavior due to its solitary, immersive, and algorithm-driven nature [18]. This environment fosters higher levels of impulsivity [19] and prolonged engagement [20], often without immediate awareness of financial or psychological harm. Several studies have shown that individuals who primarily gamble online tend to exhibit more severe symptoms of disordered gambling, including increased cravings [21], loss of control [22], and difficulty abstaining [23]. Despite this, much of the existing research still focuses on generalized gambling behavior, without distinguishing the unique cognitive and emotional dynamics associated with internet-based gambling. Consequently, there is a growing need for experimental paradigms that capture the specific behavioral and physiological responses elicited by digital gambling environments.

### 1.3. Neurophysiological and Psychophysiological Correlates

Neurobiological models of addiction have increasingly emphasized the importance of cue reactivity—heightened physiological and cognitive responses to addiction-related stimuli [24]—as a central mechanism sustaining compulsive behaviors [25]. In the context of Gambling Disorder, exposure to gambling-related cues such as slot machines [26], betting slips [27], or sports scores [28] can elicit rapid neurophysiological and autonomic responses that mirror those seen in substance use disorders [29]. Event-related potentials (ERPs), particularly the P300 and LPP, have been widely used to index attentional allocation and emotional engagement in response to addiction-relevant stimuli [30]. The P300, typically observed 300–400 ms post-stimulus, reflects orienting and salience detection, while the LPP, emerging around 500–800 ms, is associated with sustained motivational relevance [31]. Both have been shown to be amplified in individuals with behavioral or substance addictions when exposed to disorder-specific cues [32].

In parallel, GSR measures such as SCL and SCR offer robust indicators of autonomic arousal and emotional intensity [33]. Studies have demonstrated that individuals with Gambling Disorder exhibit increased phasic and tonic skin conductance activity in response to win or loss-related cues [34,35], supporting the role of sympathetic arousal in gambling motivation.

Moreover, theta-band activity has been linked to cognitive engagement and capacity [36], to combined cognitive and emotional engagement during demanding interpersonal tasks [37], to flexible switching between emotion regulation strategies [38], and to learning under threat and uncertainty [39]. Notably, several of these effects have been observed at lateral frontopolar and frontocentral sites rather than at the midline [38,39], indicating that theta-band indices of engagement are not confined to midline recordings. Because gambling outcomes—and near-misses in particular—combine reward ambiguity with a demand for behavioral adjustment, theta activity is a plausible candidate marker of cue reactivity in this context. Evidence in gambling populations specifically remains limited, however, and no directional prediction was formulated in the present study.

Despite these findings, most studies investigating neurophysiological and psychophysiological markers in gambling rely on static or scripted cue presentations (e.g., images, sounds, video clips), limiting their ecological validity. There remains a critical gap in understanding how these markers behave in more immersive, dynamic, and interactive gambling contexts—especially in populations whose primary gambling behavior occurs online.

### 1.4. Role and Advantages of Virtual Reality in Addiction Research

Virtual reality (VR) provides an ecologically valid and interactive platform for studying addictive behaviors by simulating realistic, multisensory environments under controlled experimental conditions [40]. Compared with traditional two-dimensional cue paradigms, VR enables participants to engage with addiction-related stimuli in real time, thereby enhancing emotional salience and eliciting more naturalistic behavioral and physiological responses [41]. In substance use disorders, VR has demonstrated utility for eliciting craving, assessing attentional biases, and evaluating cue-exposure interventions [42].

The application of VR in the context of Gambling Disorder is particularly promising [27]. Casino-like settings [43]—complete with ambient noise, visual stimuli, and user-controlled betting mechanisms [44]—can be faithfully replicated, offering a controlled yet realistic platform for examining cognitive and affective processes related to gambling. When combined with neurophysiological measures such as EEG and psychophysiological markers like GSR, VR paradigms allow researchers to probe cue reactivity and decision-making in a context that resembles real-world gambling far more closely than image-based or scripted alternatives. Additionally, VR allows for the manipulation of specific gambling scenarios (e.g., wins, losses, near-misses) under standardized conditions, offering new insights into the neural and behavioral correlates of pathological gambling, particularly among individuals whose engagement is predominantly online [45].

### 1.5. Research Gap and Rationale

Although previous studies have investigated cue reactivity in Gambling Disorder using neurophysiological and psychophysiological measures, most have relied on static cue paradigms or examined neural and autonomic responses separately, limiting their ability to capture cue reactivity during realistic gambling experiences. Although VR has been applied in addiction research [46,47], few studies have combined immersive VR with simultaneous EEG and GSR recordings in gambling research [48]. Furthermore, relatively few studies have specifically examined individuals with Internet Gambling Disorder [49,50] or compared their neural, autonomic, and behavioral responses during standardized gambling outcomes such as wins, losses, and near-misses.

To address this gap, the present study combined immersive VR with simultaneous EEG and GSR recordings to examine attentional, emotional, and autonomic responses to gambling cues under standardized but realistic conditions. This approach provides a more ecologically valid framework for investigating cue reactivity in individuals with Internet Gambling Disorder.

### 1.6. Study Aims and Hypotheses

The primary objective of this study was to examine the neural, autonomic, and behavioral mechanisms underlying cue reactivity in individuals with Internet Gambling Disorder (IGD) within an immersive virtual reality (VR) gambling environment. To achieve this, electroencephalography (EEG), galvanic skin response (GSR), and behavioral performance measures were collected simultaneously while participants completed a standardized 20 min VR casino task incorporating predetermined win, loss, and near-miss outcomes. This multimodal approach was designed to provide an ecologically valid assessment of gambling cue reactivity under conditions that closely resemble real-world online gambling.

We hypothesized that individuals with Internet Gambling Disorder would exhibit stronger neural, autonomic, and behavioral responses to gambling-related cues than healthy controls. Specifically, the IGD group was expected to demonstrate larger P300 and late positive potential (LPP) amplitudes, reflecting enhanced attentional allocation and sustained emotional processing of gambling-related stimuli. Greater autonomic reactivity was also expected, as indicated by higher skin conductance level (SCL) and skin conductance response (SCR) measures. Finally, participants with IGD were expected to exhibit greater behavioral engagement during the VR task and report higher levels of post-task gambling craving than healthy controls. In addition to these confirmatory hypotheses, frontal theta power was examined on an exploratory basis. Because evidence for theta-band activity in Gambling Disorder is limited and no directional prediction could be justified a priori, this measure was analyzed without a specified hypothesis and its results are interpreted accordingly.

## 2. Method

### 2.1. Study Design

This study employed a between-subjects experimental design to examine neurophysiological, autonomic, and behavioral responses to gambling-related cues in individuals diagnosed with Gambling Disorder—predominantly through online platforms—compared to healthy controls. Participants were immersed in a standardized, pre-scripted VR casino environment that included slot machines, poker tables, and sports betting interfaces. All gambling outcomes were controlled and identical across participants to ensure experimental consistency and eliminate outcome variability as a confound.

Neurophysiological activity was recorded via wireless EEG, while autonomic arousal was measured through GSR. Behavioral data related to decision-making and risk-taking were collected in real time during the VR task. The design enabled temporal synchronization of EEG, GSR, and behavioral events through embedded digital triggers. This integrative, multimodal approach allowed for the precise examination of cue reactivity, emotional engagement, and cognitive control mechanisms in Internet Gambling Disorder.

### 2.2. Sample Size Estimation

A priori power analysis was conducted using G*Power (version 3.1.9.7) to determine the required sample size for detecting medium-to-large effect sizes in repeated-measures designs involving group-by-condition interactions. Based on previous psychophysiological studies examining cue reactivity in addiction using EEG and GSR (effect size f = 0.30), with an alpha level of 0.05 and power set at 0.80, the analysis indicated a minimum of 27 participants per group was required. To account for potential data loss due to technical issues (e.g., EEG artifact contamination, GSR signal dropout), participant noncompliance, or withdrawal from the study, the initial recruitment target was set at 30 participants per group, totaling 60 participants. This allowed for adequate statistical power while accommodating expected attrition in both clinical and control samples.

### 2.3. Participants

The study initially aimed to recruit a total of 60 participants, comprising 30 individuals with Gambling Disorder and 30 healthy controls. Recruitment for the clinical group was conducted through four private outpatient psychiatry clinics located in different districts of Istanbul—Kadıköy, Ümraniye, Beşiktaş, and Şişli—to ensure sociodemographic diversity. Professor GHS, a psychiatrist, oversaw participant identification through his own clinic and three additional collaborating clinics. A total of 47 potential participants with suspected gambling-related problems were initially screened. Clinical interviews were conducted by EY, a senior clinical psychologist, using the Structured Clinical Interview for DSM-5—Clinician Version (SCID-5-CV). Based on the interviews, 37 participants were retained, and final diagnoses were confirmed by Professor GHS. Ultimately, 32 individuals met DSM-5-TR criteria for Gambling Disorder, with a primary pattern of internet-based gambling behavior.

From the clinical group, four participants were excluded: one failed to attend the experiment despite multiple rescheduling attempts; one had consumed alcohol prior to the session, compromising EEG reliability; one declined to provide consent for publication; and one dataset was rendered unusable due to an electrical failure on campus. Consequently, the final sample for the Gambling Disorder group consisted of 28 participants. Healthy controls were recruited through academic networks at three different universities and were matched to the clinical group in terms of age, gender, and education level. Three control participants were excluded: one failed to complete the experiment, one did not show up on the experiment day, and one experienced an acute panic reaction during the VR session and was referred to psychiatric care. The final control group comprised 29 participants. All participants in the Internet Gambling Disorder group were male, consistent with the demographic distribution observed in clinical populations of online gambling users.

### 2.4. Inclusion and Exclusion Criteria

Participants in the Internet Gambling Disorder group were required to meet the diagnostic criteria for Gambling Disorder according to the DSM-5-TR, confirmed through SCID-5-CV, with online gambling identified as their primary mode of engagement. Additional inclusion criteria included: being between 18 and 55 years of age, right-handed, fluent in Turkish, and having normal or corrected-to-normal vision and hearing. Participants were required to be free of psychotropic medication use for at least four weeks prior to participation. Healthy controls were required to have no history of gambling behavior (recreational or problematic), no current or past psychiatric diagnoses, and no first-degree relatives with a history of addiction or psychiatric disorders. Controls were matched to the experimental group in terms of age, gender, and educational background.

General exclusion criteria for both groups included: presence of neurological disorders (e.g., epilepsy, head trauma), current substance use disorder, cognitive impairments, cardiovascular conditions that may influence physiological reactivity, or any contraindications for VR exposure (e.g., vertigo, motion sickness). Participants who had consumed alcohol or drugs within 24 h of the experiment or failed to provide written informed consent for data use and publication were also excluded.

### 2.5. VR Environment and Task Procedure

The virtual gambling environment was developed in Unity by an external programmer under the supervision of SVÜ (the author) and head of the Neuro Marketing Research Laboratory at Üsküdar University, where the experimental sessions were conducted. The simulation was delivered through the Oculus Quest 2 headset with integrated hand controllers. Designed to replicate a lifelike casino atmosphere, the environment featured interactive slot machines, poker tables, and a sports betting station. Although the participants primarily engaged in internet-based gambling, the virtual environment was designed to recreate the gambling activities most commonly available on contemporary online gambling platforms, including slot machines, poker, and sports betting. Rather than replicating the graphical interface of a specific gambling website or mobile application, the objective was to create an immersive and standardized gambling context that captured the core behavioral and sensory characteristics of online gambling while maintaining experimental control and compatibility with simultaneous EEG and GSR recordings. Participants navigated the space from a first-person perspective, using natural hand movements to place bets, operate slot levers, and make in-game decisions. All participants experienced an identical pre-programmed sequence of gambling events to ensure experimental consistency. The standardized sequence consisted of 18 gambling events, including 5 wins, 8 losses, and 5 near-miss outcomes, distributed across 10 slot machine spins, 5 poker rounds, and 3 sports-betting trials. The order of outcomes was predetermined and identical for all participants to ensure standardized cue exposure while preventing prolonged runs of the same outcome type. Participants were informed that they would interact with a realistic virtual gambling environment but were not informed that the gambling outcomes were predetermined, thereby maintaining the perception that outcomes were contingent on their own decisions. All outcome events were synchronized with physiological recording devices through digital triggers, enabling precise temporal alignment of EEG and GSR recordings with gambling events.

The experimental procedure consisted of three phases:

- Baseline Phase (2 min): Participants sat in a neutral VR setting with no interactive elements, allowing for baseline EEG and GSR recordings.
- Gambling Phase (20–25 min): Participants interacted with the VR casino in three blocks consisting of 10 slot machine spins, 5 poker rounds, and 3 sports-betting trials, during which the predetermined sequence of 5 wins, 8 losses, and 5 near-miss outcomes was presented. Visual and auditory feedback simulated the sensory effects of real gambling environments.
- Craving Assessment (Post-Task): At the end of the session, participants rated their urge to gamble using a Virtual Analog Scale (VAS) displayed within the VR environment. This scale ranged from 0 (“no craving at all”) to 100 (“extreme craving”) and was controlled via the VR hand interface, allowing participants to indicate their subjective craving intensity while still immersed in the simulation.

All behavioral interactions and outcome events were time-stamped and logged within the Unity platform for synchronization with EEG and GSR signals during analysis.

**Figure 1:**
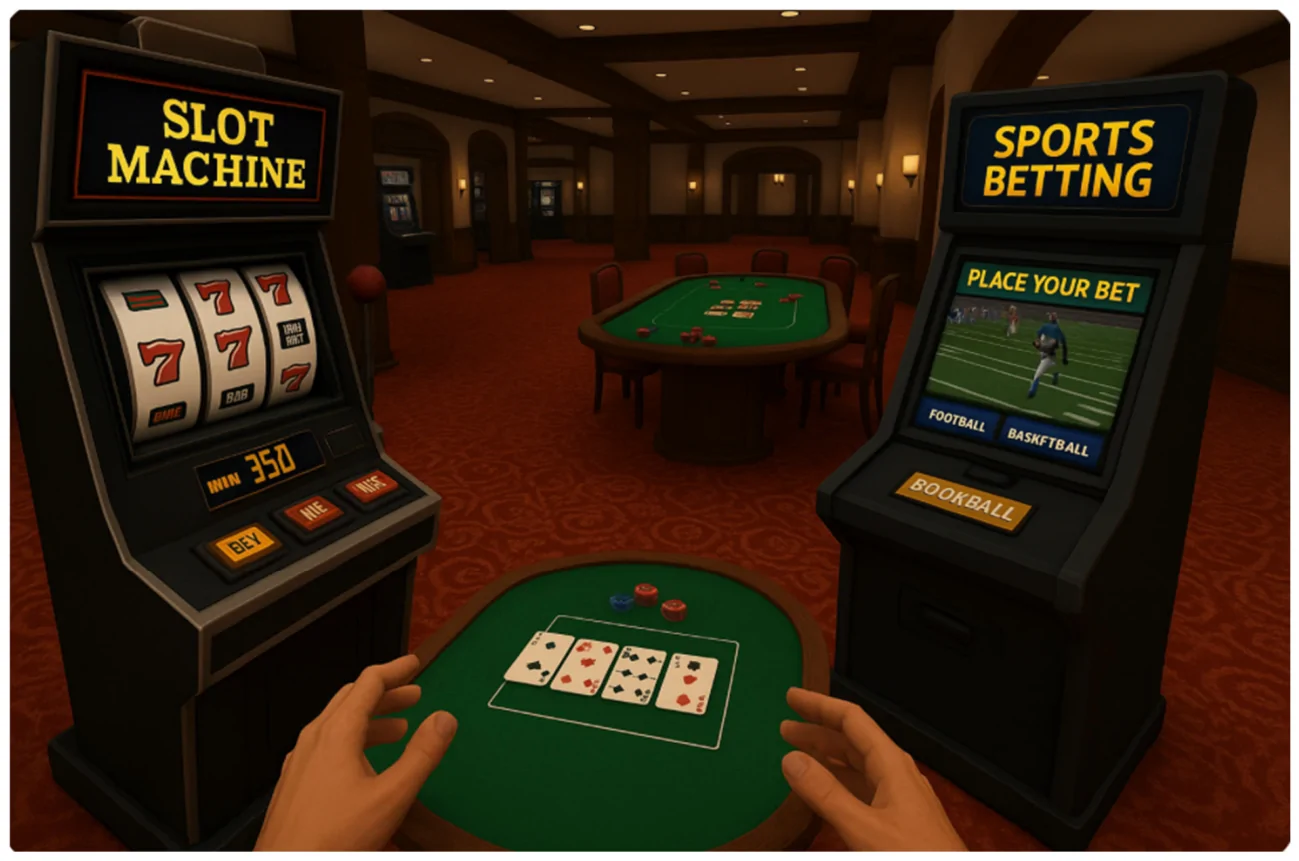
A 3D-rendered representation of the virtual reality gambling environment developed for this study. The simulation included interactive slot machines, poker tables, and a sports betting kiosk, designed in Unity and delivered via Oculus Quest 2 with hand controllers.

Figure 1 illustrates the immersive VR environment participants engaged with during the experiment. The virtual setting replicates key features of a casino, enabling users to navigate and interact with various gambling interfaces in real time, including slot machines, card tables, and sports betting stations. This digital design was intended to simulate ecologically valid gambling scenarios while standardizing exposure across participants. The realistic visuals and interactivity facilitated the collection of synchronized EEG and GSR data during gambling-related decision-making, making the VR environment a central component in evaluating cue reactivity among individuals with Internet Gambling Disorder.

### 2.6. EEG Data Acquisition and Processing

EEG activity was recorded using the Emotiv Epoc X wireless system, which features 14 semi-dry Ag/AgCl electrodes positioned according to the manufacturer’s standard montage: AF3, AF4, F3, F4, Fz, FCz, F7, F8, T7, T8, P7, P8, O1, and O2. The reference configuration followed a common-mode sense (CMS) and driven right leg (DRL) setup located near the mastoids. EEG signals were sampled at 128 Hz, with onboard hardware filtering between 0.16 and 43 Hz and a 50 Hz notch filter to eliminate power-line noise. Experimental sessions were conducted in a quiet laboratory under standardized environmental conditions, with consistent room temperature and ambient lighting across participants. Participants were instructed to minimize unnecessary body and hand movements throughout the recording to reduce motion-related artifacts while remaining comfortably seated during the VR task. Stimulus onset markers were embedded within the Unity-based VR environment using digital triggers to ensure precise synchronization between gambling events and EEG recordings. Offline preprocessing was performed in iMotions 10.0.1. Signals were re-referenced to the mastoid averages and band-pass filtered between 0.1 and 30 Hz using a finite impulse response (FIR) filter. Continuous EEG data were segmented into epochs extending from −200 ms to 800 ms relative to gambling outcome onset. Baseline correction was performed using the −200 to 0 ms pre-stimulus interval. Artifact detection combined automated algorithms with manual visual inspection, and epochs exceeding ±100 μV were rejected. Independent Component Analysis (Infomax ICA) was subsequently applied to identify and remove components associated with ocular, muscular, and movement artifacts. Approximately 7.4% of epochs were excluded following artifact rejection. ERP quantification was constrained by the fixed 14-channel Emotiv Epoc X montage, which does not include midline or centroparietal sites (Pz, CPz, CP1/CP2). Component measurement electrodes were therefore selected on the basis of the closest available coverage to the canonical scalp distributions of each component. The P300 was quantified at the lateral parietal sites P7 and P8, the nearest available approximation to the midline parietal maximum at which the P3b is typically largest [51]. Late positivity was quantified at O1 and O2. Although the LPP in adults is conventionally measured at centroparietal sites, temporospatial decomposition indicates that the scalp-recorded LPP comprises a distributed set of positivities with occipital, parietal, and central contributions, all of which are enhanced for motivationally significant stimuli [52,53]. The occipital measure obtained here should therefore be understood as indexing the posterior component of this distributed late positivity rather than as equivalent to a centroparietal LPP, and we refer to it throughout as posterior late positivity to make this distinction explicit. Consistent with this, the reference configuration placed CMS/DRL at the mastoids rather than at the P3/P4 positions used in some Emotiv configurations, avoiding attenuation of activity at the posterior sites of interest. Time-frequency decomposition was additionally performed to quantify frontal theta power (4–7 Hz) at lateral frontal sites, as the montage does not include midline electrodes. This measure was examined on an exploratory basis and was not subject to a priori hypotheses. The preprocessed EEG data were subsequently exported to R (version 4.3.0), where ERP extraction, time-frequency analyses, and statistical analyses were performed using open-source ERP and spectral analysis packages. EEG acquisition, preprocessing, and ERP quantification followed the reporting standards and methodological recommendations of the Society for Psychophysiological Research guidelines for ERP research [54,55] and the OHBM COBIDAS MEEG recommendations for reproducible EEG and MEG research [56]. Accordingly, we report sensor and reference locations, online and offline filter characteristics, epoch and baseline intervals, artifact-rejection criteria and rejection rates, and the electrodes and time windows used for component quantification. Offline band-pass filtering (0.1–30 Hz, FIR) was carried out using the standard preprocessing pipeline implemented in iMotions 10.0.1; because this pipeline does not expose the full filter specification (order, phase response, and roll-off) to the user, these parameters cannot be reported here. As filter characteristics can influence ERP amplitude and latency estimates [57], this is noted as a reporting limitation. Because the Emotiv Epoc X uses a fixed 14-channel montage without midline parietal coverage, P300 was quantified at the closest available posterior sites (P7, P8) and the LPP at O1 and O2 rather than at the canonical Pz/CPz and centro-parietal locations; this constraint is acknowledged in the Limitations section. Adapted Emotiv systems have nonetheless been shown to yield late ERP components, including the P3, that are comparable in morphology and amplitude to those obtained with research-grade amplifiers [58,59]. Theta power (4–7 Hz) was quantified over the post-outcome interval using the time-frequency decomposition routine implemented in iMotions 10.0.1 and averaged across frontal electrodes. Because this analysis was conducted within the software’s standard pipeline, the underlying decomposition parameters are not user-configurable and cannot be specified further here. Epoch rejection removed 7.4% of trials overall; per-condition trial counts were not retained separately by group, and the possibility that differential data loss contributed to group differences in signal-to-noise therefore cannot be fully excluded.

### 2.7. Galvanic Skin Response Acquisition and Processing

GSR data were collected using the Shimmer3 GSR+ system equipped with GSR100C amplifiers. Two isotonic, pre-gelled Ag/AgCl electrodes were affixed to the thenar and hypothenar areas of the non-dominant hand following standard skin preparation procedures. Signals were sampled at 128 Hz and recorded using AcqKnowledge 5.0 software. To ensure temporal synchronization with in-game gambling events, digital event markers embedded in the Unity environment were used to align stimulus onset with GSR signal acquisition. The data were imported into iMotions software for preprocessing, including artifact rejection, smoothing, and baseline correction. Preprocessing included signal smoothing, artifact detection, visual inspection, and decomposition into tonic and phasic components using the automated processing pipeline implemented within iMotions. Participants whose recordings did not exhibit measurable phasic skin conductance responses across the experimental session (non-responders) would have been excluded from the analyses; however, no participants met this criterion.

Tonic arousal was measured via SCL, defined as the mean signal amplitude during each gambling trial. Phasic SCRs were identified as increases in conductance equal to or greater than 0.05 µS occurring within a 1–4 s window following stimulus onset. All SCL and SCR data were visually inspected and manually corrected if necessary. The cleaned data were exported to R (version 4.3.0). Group differences in SCL and SCR were examined using independent-samples *t*-tests for condition-specific comparisons and analyses of variance for overall group and group-by-condition effects.

### 2.8. Behavioral Measures and Psychometrics

Behavioral data were collected throughout the VR gambling task and included metrics such as reaction time, bet selection, and loss-chasing behavior. All participant actions were logged in real time within the Unity environment and time-stamped for later alignment with EEG and GSR recordings. Loss-chasing was operationally defined as placing consecutive high-risk bets following multiple losses, despite decreasing virtual currency. In addition to behavioral measures, participants completed a battery of psychometric assessments prior to the VR session.

### 2.9. The Problem Gambling Severity Index

The Problem Gambling Severity Index (PGSI) was developed by Ferris and Wynne (2001) [60] to assess gambling-related problems in the general population. The scale consists of 9 items rated on a 4-point Likert scale from 0 (never) to 3 (almost always), with higher scores indicating greater gambling severity. The Turkish version was validated by Arcan (2020) [61] and demonstrated good psychometric properties (Cronbach’s α = 0.87).

### 2.10. The Structured Clinical Interview for DSM-5—Clinician Version

The Structured Clinical Interview for DSM-5–Clinician Version (SCID-5-CV) was developed by the American Psychiatric Association to assess DSM-5 psychiatric disorders, including Gambling Disorder. It is a semi-structured diagnostic interview administered by trained clinicians. The Turkish version was validated by Elbir et al. (2019) [62] and demonstrated good interrater reliability (Cohen’s κ = 0.70–0.83).

### 2.11. The Barratt Impulsiveness Scale

The Barratt Impulsiveness Scale–11 (BIS-11) was developed by Barratt (1995) [63] to assess trait impulsivity. The scale consists of 30 items rated on a 4-point Likert scale, with higher scores indicating greater impulsivity. The Turkish version was validated by Güleç et al. (2008) [64] and demonstrated good psychometric properties (Cronbach’s α = 0.80).

### 2.12. The State-Trait Anxiety Inventory

The State-Trait Anxiety Inventory (STAI) was developed by Spielberger et al. (1970) [65] to assess state and trait anxiety. The instrument consists of 40 items rated on a 4-point Likert scale, with higher scores indicating greater anxiety. The Turkish version was validated by Tomak et al. (2021) [66] and demonstrated good psychometric properties (Cronbach’s α = 0.83 for the trait subscale and 0.87 for the state subscale).

### 2.13. The Gambling Craving Scale

The Gambling Craving Scale (GACS) was developed by Young and Wohl (2009) [67] to assess gambling craving. The scale consists of 9 items rated on a 7-point Likert scale, with higher scores indicating greater craving. The Turkish version was validated by Savci and Griffiths (2019) [68] and demonstrated good psychometric properties (Cronbach’s α = 0.88–0.91).

During the final phase of the VR task, participants rated their current craving intensity using a virtual analog scale (VAS) embedded in the simulation. This in-VR scale ranged from 0 (“no craving at all”) to 100 (“extreme craving”) and was controlled via the VR hand interface. All psychometric data were linked to physiological and behavioral responses for integrated analysis.

### 2.14. Statistical Analyses

Statistical analyses were performed using R (version 4.3.0). Before hypothesis testing, the data were examined for normality, outliers, and missing values. Outlying EEG, GSR, behavioral, and craving observations exceeding ±3 standard deviations were winsorized. Descriptive statistics are presented as means and standard deviations for continuous variables and frequencies and percentages for categorical variables.

Independent-samples *t*-tests were used to compare the Internet Gambling Disorder and healthy control groups on continuous demographic, psychometric, EEG, condition-specific GSR, and behavioral outcomes. Chi-square tests were used for categorical variables. Analyses of variance were conducted where comparisons involved overall group effects or responses across multiple gambling conditions. Bonferroni-adjusted post hoc comparisons were applied when follow-up pairwise testing was required.

Pearson’s correlation coefficients were calculated within the Internet Gambling Disorder group for a targeted set of six associations, each corresponding to a distinct theoretical prediction rather than to an exhaustive exploration of the correlation matrix. Three examined convergence between constructs expected to be related: trait anxiety with late positivity to near-miss outcomes, on the premise that anxious individuals show amplified emotional reactivity to ambiguous, reward-proximal events; gambling craving with the number of bets initiated, as a behavioral validity check on the VR paradigm; and near-miss electrodermal reactivity with gambling craving, testing whether autonomic cue reactivity tracks subjective urge. Two examined trait impulsivity in relation to frontal theta power and to state craving, testing whether a dispositional measure maps onto cognitive-control and motivational indices respectively. The sixth, trait anxiety with electrodermal responses to loss outcomes, was included as a discriminant comparison: if the association between anxiety and reactivity is specific to ambiguous outcomes, no comparable relationship should be expected for unambiguous losses.

Two multiple linear regression models were conducted within the Internet Gambling Disorder group, with predictors selected to represent distinct construct domains rather than to maximize explained variance, and limited in number given the available sample. The first model predicted the number of bets initiated from gambling craving (motivational), frontal theta power (cognitive-control, entered as an exploratory predictor), and trait anxiety (affective), testing whether behavioral engagement is driven principally by motivational rather than cognitive or affective factors. The second model predicted post-task subjective craving from near-miss electrodermal reactivity and trait impulsivity, contrasting a state-level physiological predictor with a trait-level dispositional one. Predictors within each model were selected to be conceptually non-redundant, and models were deliberately restricted to three and two predictors respectively to maintain an acceptable ratio of cases to parameters at *n* = 28.

Effect sizes were reported as Cohen’s d for independent-samples *t*-tests, partial eta squared (η^2^p) for analyses of variance, Pearson’s r for correlations, and R^2^ for regression models. Bonferroni correction was applied to post hoc pairwise comparisons where appropriate. Because the remaining confirmatory analyses addressed predefined, hypothesis-driven outcomes, no global adjustment was applied. Analyses involving frontal theta power were exploratory and are reported as such rather than as confirmatory tests.

## 3. Results

Table 1 summarizes the demographic and psychometric characteristics of the participants. The groups did not differ significantly in age or years of education. Participants with Internet Gambling Disorder had significantly higher gambling-severity scores on the PGSI, higher trait anxiety scores on the STAI-T, higher trait impulsivity scores on the BIS-11, and higher gambling-craving scores on the GACS than healthy controls.

Table 2 presents the EEG, physiological, and behavioral outcomes comparing participants with Gambling Disorder and healthy controls across win, loss, and near-miss conditions. P300 amplitudes during win trials were significantly higher in the gambling group compared to controls (*t*(55) = 2.31, *p* = 0.025), reflecting enhanced attentional allocation to reward cues. However, no significant differences emerged for P300 amplitudes during loss or near-miss conditions, suggesting a condition-specific effect.

LPP amplitudes revealed a more pronounced pattern. While win and loss trials did not differ significantly between groups, near-miss trials elicited significantly greater LPP amplitudes in the gambling group (*t*(55) = 3.02, *p* = 0.004), indicating heightened emotional engagement in response to ambiguous outcomes. Time-frequency analysis of frontal theta power during decision phases showed numerically higher values in the gambling group; however, the group difference was not statistically significant (*t*(55) = 1.61, *p* = 0.113). Therefore, no conclusions can be drawn regarding between-group differences in frontal theta activity.

Physiologically, SCL was significantly elevated in the gambling group across conditions (*F*(1,55) = 7.12, *p* = 0.009), consistent with generalized autonomic arousal. In contrast, SCRs to near-miss outcomes were selectively heightened in the gambling group (*t*(55) = 2.45, *p* = 0.018), supporting specific cue-reactivity to near wins.

Behaviorally, gambling participants showed greater task engagement, as evidenced by a higher number of interactions and longer betting durations (*t*(55) = 3.51, *p* = 0.001). Post-task subjective craving, measured using the visual analog scale (VAS), was significantly higher in the gambling group (*t*(55) = 4.62, *p* < 0.001), while remaining stable in controls. These findings validate the VR paradigm’s ecological validity and its capacity to elicit differential neural and physiological responses in individuals with Gambling Disorder.

As shown in Table 3, tonic SCL was significantly higher in the Internet Gambling Disorder group during win conditions, indicating elevated baseline autonomic arousal compared to controls (*F*(1,55) = 7.12, *p* = 0.009). However, the group-by-condition interaction did not reach significance for near-miss events (*F*(2110) = 1.88, *p* = 0.157), suggesting that although overall SCL was higher among gamblers, the pattern did not vary meaningfully across outcome types. No specific differences were found in SCL between groups during loss trials.

Regarding phasic reactivity, SCRs to near-miss outcomes were significantly larger in the gambling group than in controls (*t*(55) = 2.45, *p* = 0.018), pointing to a distinct sensitivity to ambiguous, reward-like cues. In contrast, no significant group differences were observed in SCR amplitude during win or loss trials (*p* > 0.29). This pattern indicates that near-miss stimuli uniquely trigger enhanced sympathetic nervous system responses in individuals with gambling problems, even though general autonomic responses (SCL) remain elevated across conditions.

**Figure 2:**
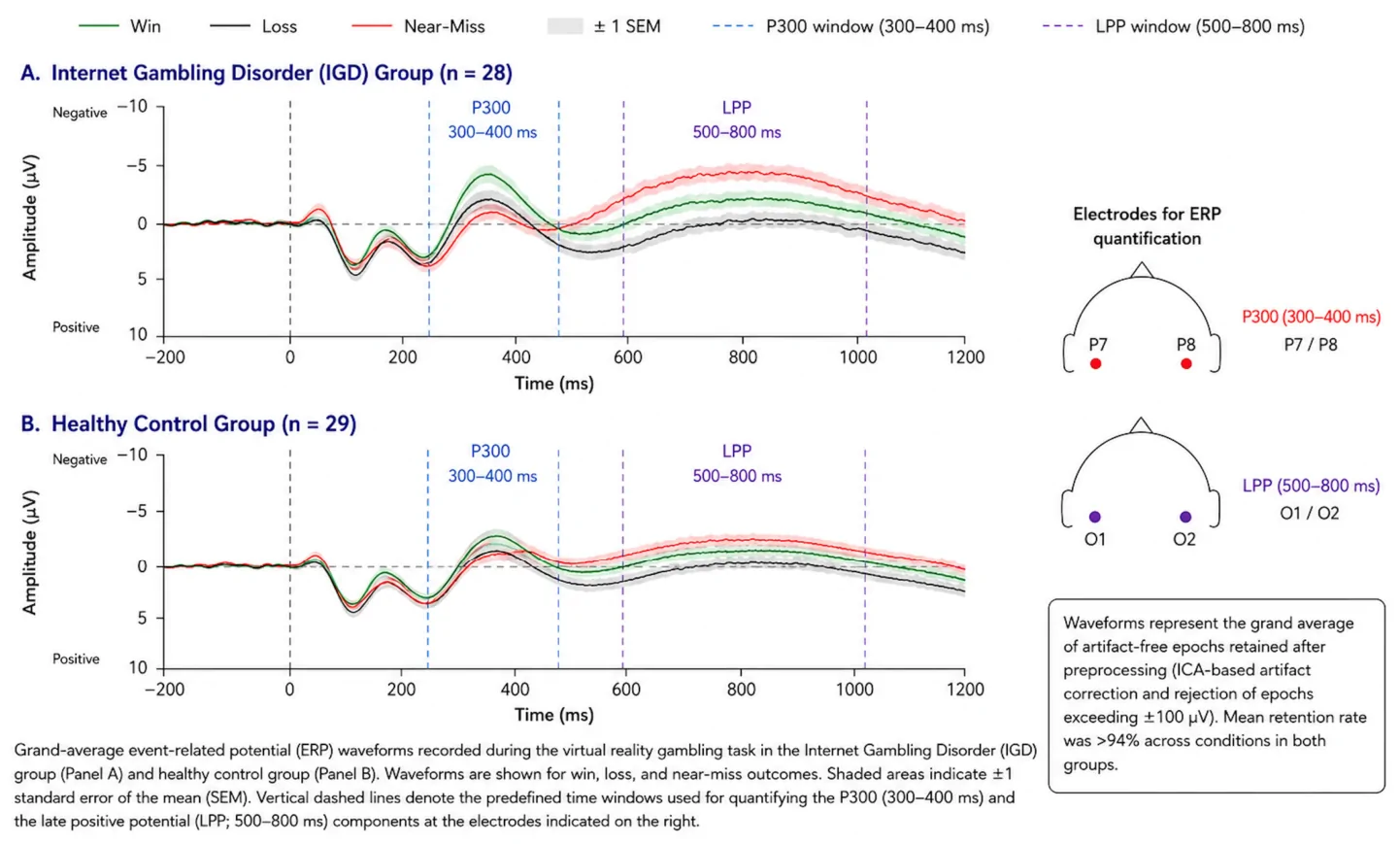
ERP and SCR responses to gambling cues in the VR task.

As shown in Figure 2, Grand-average event-related potential waveforms during the virtual reality gambling task, averaged across the four posterior electrodes used for component quantification (P7, P8, O1, O2), for the Internet Gambling Disorder group (Panel A, *n* = 28) and the healthy control group (Panel B, *n* = 29). Waveforms are shown separately for win, loss, and near-miss outcomes, time-locked to outcome onset and baseline-corrected to the −200 to 0 ms interval. Shaded regions indicate ±1 SEM. This pooled display is provided for descriptive purposes only; the P300 and posterior late positivity reported in Table 2 were quantified separately at P7/P8 and O1/O2 respectively, and are not read from the waveform shown here.

As shown in Table 4, the Internet Gambling Disorder group spent significantly more time in the VR gambling environment and initiated more bets than the control group, indicating heightened behavioral engagement. However, no significant group difference was observed in the average latency between gambling trials. Subjective craving scores were significantly elevated among gamblers, consistent with their increased physiological and attentional reactivity to gambling cues throughout the session.

As shown in Table 5, key correlations emerged primarily within the Internet Gambling Disorder group. Trait anxiety showed a moderate, statistically significant association with enhanced emotional reactivity to near-miss events as reflected in LPP amplitudes. Craving intensity was also significantly related to the number of bets placed, reinforcing the behavioral relevance of subjective urge. Although not all correlations reached significance, near-threshold effects—such as SCRs predicting craving—point toward nuanced psychophysiological patterns that may become clearer in larger samples. This selective significance pattern underscores the multifaceted nature of gambling addiction, where not all variables are linearly coupled but interact in complex, condition-dependent ways.

As shown in Table 6, two separate regression models were conducted to explore predictors of gambling engagement and subjective craving. In the first model, which aimed to predict the number of bets initiated during the VR gambling task, gambling craving (GACS) emerged as a significant predictor, indicating that individuals with higher craving levels tended to gamble more frequently in the virtual setting. Frontal theta activity showed a non-significant trend, although this difference was not statistically significant and therefore should not be interpreted as evidence of altered cognitive engagement or overload, while trait anxiety (STAI-T) did not significantly contribute, implying a more distal or indirect role in gambling behavior.

In the second model, subjective craving was predicted by physiological arousal and trait impulsivity. SCR amplitude in response to near-miss outcomes significantly predicted craving intensity, reinforcing the role of autonomic reactivity in reinforcing gambling urges. However, BIS-11 scores, reflecting trait impulsivity, did not reach significance, suggesting that subjective craving in the moment may be more strongly influenced by bodily responses than by pre-existing cognitive schemas.

Together, these regression models highlight that while psychological traits and cognitive beliefs are relevant, the strongest immediate predictors of gambling behavior appear to be subjective craving and physiological arousal—especially in response to emotionally charged gambling cues like near misses. This pattern supports a model of Gambling Disorder in which affective and embodied responses are primary drivers of engagement.

## 4. Discussion

The current study investigated neural, autonomic, and behavioral responses to gambling-related stimuli in individuals with Internet Gambling Disorder (IGD) using an immersive virtual reality paradigm integrated with EEG and GSR recordings. Overall, the findings were consistent with our hypotheses, showing that participants with IGD exhibited significantly larger P300 and late positive potential (LPP) amplitudes in response to gambling cues than healthy controls. These results indicate enhanced attentional processing and prolonged emotional engagement with gambling-related stimuli. Previous studies have consistently identified these ERP components as important neurophysiological markers of cue reactivity across both substance-related and behavioral addictions [69,70], and the present findings extend this evidence to a realistic virtual gambling setting. Furthermore, skin conductance responses were significantly greater in the IGD group, particularly following near-miss events, suggesting heightened autonomic arousal during these outcomes. This pattern supports the view that near-miss experiences possess strong motivational value and may contribute to the persistence of gambling behavior despite the absence of an actual reward.

The observed elevations in P300 and LPP amplitudes among the IGD group align with existing models of addiction that emphasize heightened attentional capture and emotional salience of disorder-relevant cues. P300, often interpreted as reflecting orienting and resource allocation, was particularly pronounced during reward-related outcomes, suggesting that individuals with IGD may experience increased anticipatory processing in response to gambling cues [71]. The LPP, which reflects sustained emotional engagement, was most elevated in the near-miss condition—a pattern that supports the well-documented role of near-miss events in reinforcing continued gambling behavior despite objective loss [72]. These neurophysiological findings mirror prior EEG studies in substance and behavioral addictions [73], which show similar reactivity to salient, personally relevant stimuli. However, our use of an immersive, first-person VR casino environment arguably offers a more ecologically valid platform for capturing cue-reactive dynamics, potentially bridging the gap between lab-based ERP research and real-world addictive behavior.

Furthermore, the SCR results complement the ERP findings, demonstrating elevated sympathetic arousal in the gambling group across most outcome types, with a peak in near-miss trials. This supports the conceptualization of near-misses as “almost wins” that hijack reward circuitry, despite their objective outcome [74]. The fact that the control group showed relatively muted SCRs in all conditions further underscores the disorder-specific nature of the physiological reactivity. The convergence of neural and autonomic markers highlights the potential value of multimodal psychophysiological measures for improving understanding of cue reactivity in Gambling Disorder. Although these findings are encouraging, further longitudinal and validation studies are required before such measures can be considered candidate biomarkers or incorporated into clinical assessment.

A further consideration concerns the substantial baseline difference in trait anxiety between groups (*d* = 1.60), which exceeded the magnitude of any neurophysiological or autonomic effect observed here. Trait anxiety is commonly elevated and frequently comorbid in Gambling Disorder, so this difference is unlikely to represent incidental sampling variation and is more plausibly part of the clinical picture. It nonetheless raises the question of whether the present group differences reflect gambling-specific cue reactivity or a more general disposition toward heightened emotional reactivity. Two observations bear on this. First, within the Internet Gambling Disorder group, trait anxiety was moderately associated with late positivity to near-miss outcomes (*r* = 0.39) but not with electrodermal responses to losses (*r* = 0.11), indicating a relatively selective relationship with the processing of ambiguous, reward-proximal outcomes rather than uniform amplification across conditions. Second, trait anxiety did not predict behavioral engagement in the VR task once craving was accounted for (β = 0.18, *p* = 0.204), suggesting that anxiety was not driving gambling behavior in this sample even where it was associated with neural reactivity. Taken together, these patterns are more consistent with trait anxiety moderating emotional reactivity to near-misses than with it accounting for the group differences observed. This interpretation remains provisional. Because trait anxiety and Gambling Disorder status are substantively confounded in this sample, statistical adjustment cannot cleanly separate their contributions [75], and the correlational findings derive from a single group of 28 participants without correction for multiple comparisons. Designs that stratify or match on trait anxiety, or that include an anxious non-gambling comparison group, would be needed to resolve the question.

In terms of behavioral outcomes, individuals with Internet Gambling Disorder demonstrated greater engagement within the virtual gambling environment, spending more time interacting with the task, initiating more bets, and reporting significantly higher post-task craving than healthy controls. These findings are consistent with heightened motivational engagement and increased cue reactivity during exposure to gambling-related stimuli within the immersive VR environment.

The craving scores, as measured immediately post-exposure to the VR gambling scenario, were also markedly elevated in the gambling group. This finding reinforces the idea that immersive cue exposure in a dynamic, interactive VR setting can effectively trigger psychological craving responses. Importantly, the inclusion of a pre-programmed betting sequence allowed us to control the emotional and cognitive impact of specific gambling outcomes (e.g., win, loss, near-miss) across participants, enhancing internal validity. The consistent increase in craving following exposure suggests that immersive VR paradigms may provide a useful experimental platform for investigating cue reactivity and evaluating cue-exposure approaches in future intervention studies.

## 5. Limitations

Several limitations should be considered when interpreting the present findings. First, the clinical group consisted exclusively of male participants, whereas the healthy control group included one female participant. Although the groups did not differ significantly in sex distribution, the predominance of male participants and the slight imbalance between groups should be considered when interpreting the findings, as sex differences have been reported in neural and autonomic responses to emotionally salient stimuli. Consequently, the generalizability of the findings to women and gender-diverse individuals remains limited. Future studies should recruit larger and more sex-balanced samples to examine potential sex-related differences in cue reactivity associated with Internet Gambling Disorder.

Second, the standardized sequence of gambling outcomes ensured that all participants were exposed to identical win, loss, and near-miss events, thereby improving experimental control. However, this level of standardization inevitably reduced the ecological validity of the VR paradigm. In real gambling settings, personal choice, perceived control, and decision-making autonomy are central features that influence emotional and cognitive responses. Consequently, the present design may not fully capture phenomena such as the illusion of control, expectancy, or other cognitive biases that emerge during self-directed gambling. Future VR paradigms incorporating both standardized and participant-driven gambling decisions may provide a more realistic representation of gambling behavior.

Another limitation relates to the recruitment strategy. Participants with Internet Gambling Disorder were recruited from psychiatric outpatient clinics, whereas healthy controls were recruited through university networks. Although the groups were matched on age, sex, and educational level, recruitment from different source populations may have introduced selection bias or unmeasured differences unrelated to gambling status, such as health-seeking behavior, socioeconomic characteristics, or broader psychological functioning. Future studies should aim to recruit control participants from the same or more comparable source populations to minimize potential sampling bias.

The healthy control group consisted of individuals without any history of gambling, providing a clear contrast with participants with Internet Gambling Disorder. Although this design reduced potential confounding associated with prior gambling exposure, including recreational gamblers without Gambling Disorder as an additional comparison group may have provided greater insight into the continuum from recreational gambling to clinically significant gambling problems. Future studies should consider incorporating both recreational and non-gambling control groups.

Although the primary analyses were hypothesis-driven, the large number of statistical comparisons increases the possibility of Type I error. Therefore, the findings should be interpreted cautiously and replicated in larger independent samples. Frontal theta power was examined without an a priori hypothesis, and was quantified at lateral frontal rather than midline sites; findings involving this measure should therefore be regarded as exploratory and are not directly comparable with studies measuring frontal midline theta at Fz or FCz.

An additional limitation concerns the relatively modest sample size in relation to the number of neurophysiological, autonomic, behavioral, correlational, and regression analyses performed. Although an a priori power analysis indicated that the target sample size was sufficient to detect medium-to-large effects for the primary comparisons, the study may have been underpowered for some secondary and exploratory analyses. Consequently, nonsignificant findings should be interpreted cautiously because they may reflect limited statistical power, while significant findings warrant replication in larger, independent samples to confirm their robustness.

Although the VR environment reproduced the gambling activities commonly available on online gambling platforms, it did not replicate the visual interface of a specific online gambling website or mobile application. Future studies could develop VR paradigms that more closely emulate contemporary online gambling interfaces to further enhance ecological validity.

A further limitation concerns spatial resolution and electrode coverage. The Emotiv Epoc X provides 14 fixed channels without midline or centroparietal coverage, so neither the P3b nor the LPP could be measured at the sites where each is conventionally maximal. The components reported here are best interpreted as lateral parietal and posterior indices of the underlying positivities rather than as direct equivalents of canonically measured P300 and LPP amplitudes, and absolute amplitude values are not directly comparable with studies using high-density research-grade montages. Replication with a montage including Pz and CPz is needed to establish whether the group differences observed here hold at the sites where these components are largest.

Finally, although Gambling Disorder diagnoses were established using the SCID-5-CV and confirmed through structured clinical evaluation, several factors that may influence neurophysiological and autonomic responses were not systematically assessed or controlled. These include psychiatric comorbidities such as depression and attention-deficit/hyperactivity disorder, which were not formally diagnosed; although trait anxiety was assessed using the STAI-T and differed significantly between groups, clinical anxiety disorders were not evaluated. Because each of these variables may affect EEG activity, autonomic arousal, and cue reactivity, their potential influence cannot be excluded. Future studies should incorporate more comprehensive clinical and behavioral assessments and consider statistically controlling for these potential confounders to better isolate gambling-specific effects. Moreover, EEG data were acquired using the Emotiv Epoc X, a portable wireless EEG system that offers practical advantages for immersive VR research, including ease of setup, participant comfort, and reduced movement constraints. However, its relatively low sampling rate (128 Hz) and limited 14-channel electrode montage provide lower spatial resolution and reduced signal fidelity than high-density research-grade EEG systems. Although previous studies have demonstrated the utility of the Emotiv Epoc X for ERP research, these technical characteristics may have limited the precision of ERP component estimation and restricted more detailed spatial analyses. Future studies should seek to replicate the present findings using higher-density EEG systems with greater spatial coverage and higher sampling rates.

## 6. Conclusions

Taken together, the present study provides a multimodal characterization of Internet Gambling Disorder by integrating behavioral, neurophysiological, and autonomic responses within an immersive virtual reality environment. Individuals with Internet Gambling Disorder exhibited enhanced P300 and late positive potential (LPP) amplitudes, increased skin conductance responses, and greater subjective craving during exposure to gambling-related cues, particularly following near-miss outcomes. These findings contribute to the growing literature on cue reactivity in behavioral addictions and support the potential value of combining virtual reality with EEG and autonomic measures to investigate the mechanisms underlying problematic gambling.

Although these findings are encouraging, they should be considered preliminary. The relatively modest sample size, predominantly male sample, use of a consumer-grade EEG system, and cross-sectional design limit the generalizability and clinical interpretation of the results. Consequently, the present findings should not be interpreted as establishing clinical biomarkers or assessment tools. Future studies should replicate these findings in larger and more diverse samples, employ higher-density research-grade EEG systems, and incorporate longitudinal and prospective designs to determine the reliability, generalizability, and potential clinical relevance of these neurophysiological and autonomic measures.

## Figures and Tables

**Table 1:** Sample characteristics of the Internet Gambling Disorder and control groups. Note. PGSI = Problem Gambling Severity Index; STAI-T = State-Trait Anxiety Inventory–Trait subscale; GACS = Gambling Craving Scale. The SCID-5-CV was used to establish Gambling Disorder diagnoses and was not treated as a continuous outcome measure.

Variable	Internet Gambling Disorder (*n* = 28)	Control Group (*n* = 29)	Test Statistic	*p*-Value
Age (Mean ± SD)	30.2 ± 6.7	29.8 ± 6.3	*t*(55) = 0.24	0.810
Gender (% Male)	100% (*n* = 28)	89.7% (*n* = 26)	χ^2^(1) = 2.14	0.143
Education (Years ± SD)	15.6 ± 2.1	15.9 ± 2.3	*t*(55) = −0.52	0.603
PGSI Total Score (0–27)	16.7 ± 2.4	3.1 ± 1.8	*t*(55) = 26.2	<0.001
STAI Trait Anxiety (20–80)	51.3 ± 8.2	38.4 ± 7.9	*t*(55) = 6.23	<0.001
BIS-11 Total Score (30–120)	63.1 ± 9.4	53.2 ± 8.7	*t*(55) = 3.4	<0.001
GACS Total Score	31.9 ± 5.7	9.2 ± 4.1	*t*(55) = 18.7	<0.001

**Table 2:** EEG, physiological, and behavioral results.

Measure	IGD (*n* = 28)	Control (*n* = 29)	Test Statistic	*p*	Effect Size
P300 Amplitude (Win)	6.3 ± 1.0	5.1 ± 1.2	*t*(55) = 2.31	0.025	d = 0.61
P300 Amplitude (Loss)	5.2 ± 1.4	4.9 ± 1.3	*t*(55) = 0.83	0.411	d = 0.22
P300 Amplitude (Near-Miss)	5.3 ± 1.5	5.0 ± 1.4	*t*(55) = 1.11	0.272	d = 0.29
LPP Amplitude (Win)	6.5 ± 1.0	6.2 ± 1.1	*t*(55) = 1.02	0.312	d = 0.27
LPP Amplitude (Loss)	6.2 ± 1.1	6.0 ± 1.2	*t*(55) = 0.61	0.544	d = 0.16
LPP Amplitude (Near-Miss)	7.2 ± 1.0	6.1 ± 1.3	*t*(55) = 3.02	0.004	d = 0.80
Frontal Theta	2.9 ± 0.7	2.3 ± 0.8	*t*(55) = 1.61	0.113	d = 0.43
SCL	3.7 ± 0.4	3.1 ± 0.5	*F*(1,55) = 7.12	0.009	η^2^ = 0.11
SCR (Near-Miss)	0.18 ± 0.05	0.10 ± 0.03	*t*(55) = 2.45	0.018	d = 0.65
VR Task Engagement	12.1 ± 2.3	8.4 ± 2.1	*t*(55) = 3.51	0.001	d = 0.93
Craving (Post-VR)	31.8 ± 4.5	24.6 ± 3.2	*t*(55) = 4.62	<0.001	d = 1.22

**Table 3:** GSR results: SCL and SCR across conditions.

Measure	IGD (*n* = 28)	Control (*n* = 29)	Test Statistic	*p*	Effect Size
Time-on-Task	17.6 ± 1.8	14.8 ± 2.1	*t*(55) = 3.51	0.001	d = 0.93
Number of Bets Initiated	25.7 ± 5.3	18.2 ± 4.6	*t*(55) = 4.02	<0.001	d = 1.06
Trial-to-Trial Latency	2.4 ± 0.6	2.3 ± 0.5	*t*(55) = 1.13	0.267	d = 0.30
Subjective Craving (VAS)	36.9 ± 10.7	12.6 ± 8.3	*t*(55) = 4.62	<0.001	d = 1.22

**Table 4:** Behavioral task performance and subjective craving scores.

Measure	Control	IGD	Test Statistic	*p*	Effect Size
Time-on-Task	14.8 ± 2.1	17.6 ± 1.8	*t*(55) = 3.51	0.001	d = 0.93
Number of Bets Initiated	18.2 ± 4.6	25.7 ± 5.3	*t*(55) = 4.02	<0.001	d = 1.06
Trial-to-Trial Latency	2.3 ± 0.5	2.4 ± 0.6	*t*(55) = 1.13	0.267	d = 0.30
Subjective Craving (VAS)	12.6 ± 8.3	36.9 ± 10.7	*t*(55) = 4.62	<0.001	d = 1.22

**Table 5:** Pearson Correlations among psychometric, behavioral, and physiological variables within the Internet Gambling Disorder group. Note. Correlations are two-tailed. STAI-T = State–Trait Anxiety Inventory–Trait subscale; GACS = Gambling Craving Scale; BIS-11 = Barratt Impulsiveness Scale-11; LPP = late positive potential; SCR = skin conductance response; VAS = visual analog scale.

Variables	r	*p*-Value
STAI-T × LPP (Near-Miss)	0.39	0.034
GACS × Number of Bets	0.47	0.009
BIS-11 × Frontal Theta	0.21	0.183
SCR (Near-Miss) × GACS	0.31	0.062
BIS-11 × Subjective Craving (VAS)	0.18	0.247
STAI-T × SCR (Loss)	0.11	0.451

**Table 6:** Multiple linear regression models predicting gambling engagement and craving.

Predictor	β	SE	t	*p*
Model 1. Number of Bets Initiated (DV) *F*(3, 24) = 4.96, *p* = 0.008, R^2^ = 0.38.				
GACS (Craving)	0.43	0.15	2.78	0.011
Frontal Theta Power	0.26	0.14	1.74	0.091
STAI-Trait	0.18	0.13	1.29	0.204
Model 2. Subjective Craving Score (DV) *F*(2, 25) = 3.21, *p* = 0.046, R^2^ = 0.21.				
SCR (Near-Miss Condition)	0.38	0.16	2.38	0.028
BIS-11	0.19	0.15	1.26	0.219

## Data Availability

The data presented in this study are available from the corresponding author upon reasonable request due to participant privacy and ethical restrictions.
